# A rare occurrence of giant dermoid cyst in the left ischiorectal fossa in a 25-year-old female patient: a case report

**DOI:** 10.1590/0102-672020260000046e1975

**Published:** 2026-10-05

**Authors:** C S VIDYA, Manas BABU, C S Sheela DEVI, Vidya Gowdappa DODDAWAD

**Affiliations:** 1 JSS Medical College, Department of Anatomy - Mysore, Karnataka, India.; 2 JSS Medical College, Department of Pathology - Mysore, Karnataka, India.; 3 JSS Dental College and Hospital, Department of Oral Pathology and Microbiology - Mysore, Karnataka, India.

**Keywords:** Dermoid Cyst, Teratoma, Ultrasonography, Cisto Dermoide, Teratoma, Ultrassonografia

## Abstract

A dermoid cyst, also called a benign cystic teratoma, often presents as a midline mass in the trunk or sacrococcygeal region, and more commonly in the skin of the face, forehead, and scalp. Although the ischiorectal fossa contains very few structures, a wide variety of pathological entities, such as developmental cysts, epidermoid cysts, lipomas, neurofibromas, and tailgut cysts, have been reported. Dermoid cysts in the ischiorectal fossa are rare, and very few cases have been reported in the scientific literature. This case report presents an uncommon presentation of a dermoid cyst in the ischiorectal fossa in a 25-year-old female patient.

## ARTICLE HIGHLIGHTS

• Ischiorectal fossa dermoid cysts are extremely rare clinical entities presenting as indolent gluteal swellings.

• Ultrasound identified a well-defined cystic mass that mimicked a perianal abscess and was resolved through complete surgical excision.

• Histopathology confirmed a classic dermoid cyst lined by squamous epithelium with abundant keratin debris.

• Early imaging and meticulous surgical resection are critical to avoid recurrence, fistula formation, or malignant transformation.

## VISUAL ABSTRACT

**Figure:**
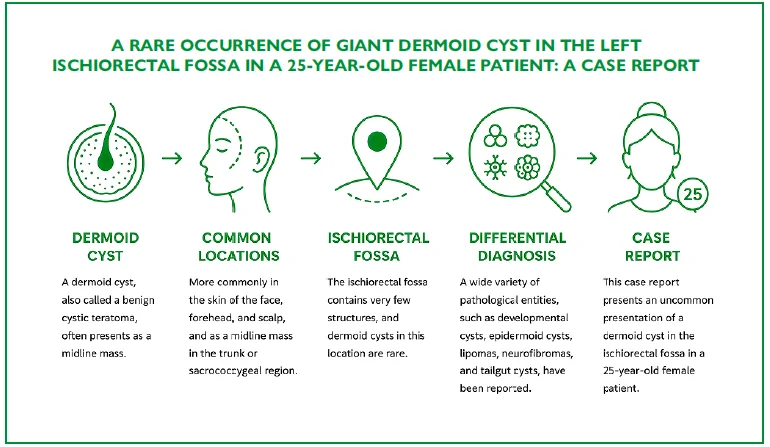

## RESUMO

Um cisto dermoide, também chamado de teratoma cístico benigno, geralmente se apresenta como uma massa na linha média do tronco ou na região sacrococcígea, e com mais frequência na pele do rosto, testa e couro cabeludo. Embora a fossa isquiorretal contenha poucas estruturas, uma ampla variedade de entidades patológicas, como cistos de desenvolvimento, cistos epidermoides, lipomas, neurofibromas e grandes cistos caudais, já foram relatadas. Os cistos dermoides na fossa isquiorretal são raros, e muito poucos foram descritos na literatura científica. Este relato de caso apresenta uma ocorrência incomum de cisto dermoide na fossa isquiorretal em uma paciente do sexo feminino de 25 anos.

## INTRODUCTION

The ischiorectal fossa, also called the ischioanal fossa, although containing few structures, is surgically important because of its great tendency to become infected. It is important to note that the ischiorectal fossae on either side communicate with each other behind the anal canal; therefore, any infection in one can readily spread to the other. Infection of this space may occur from boils or abrasions of the perianal skin, lesions within the rectum and anal canal, pelvic infection bursting through the levator ani or, rarely, via the bloodstream^9^.

Any mass in the ischiorectal fossa constitutes an eclectic group of pathologic entities. Considering the number of structures that make up this anatomical region, the variety of tumors discovered inside it is remarkable. Dermoid cysts, synonymous with benign cystic teratomas, are among the other developmental cysts, which are said to be among the most prevalent congenital abnormalities in the perirectal region^2^ ^,^ ^11^. Although most of these developmental cysts are tailgut cysts, approximately half of them are; the other common types include dermoid or epidermoid cysts, rectal duplication cysts, and neurenteric cysts^2^ ^,^ ^4^.

Although various theories have been proposed regarding the origin and development of these dermoid cysts, many authors refer to dermoid cysts as being derived from totipotent germ cells that can develop into almost any adult or fetal tissue, such as bone, hair, teeth, or muscle; and they describe well-organized tumors with well-differentiated ectodermal and mesodermal derivatives surrounding endodermal components. Dermoid cysts are uncommon, and those located in the ischiorectal region have rarely been reported.

A case of an unusually large dermoid cyst in the ischiorectal fossa in a young female patient is presented.

## METHODS

A 25-year-old woman presented with discomfort and a large swelling in the left gluteal region for one year. The swelling was initially small and gradually enlarged without pain or discharge. There was no fever associated with the swelling. On examination, a non-tender, oval-shaped, soft swelling measuring approximately 10 × 8 cm was found in the left gluteal area. The skin over the swelling was stretched because of its size but showed no signs of inflammation. During the perirectal examination, a bulge was felt in the left lateral wall of the anal canal and lower rectum.

Ultrasonography revealed a well-defined, thick-walled, encapsulated collection measuring 10.4x9x9.4 cm with homogeneous, low-level echoes, located in the left ischiorectal fossa. The collection extended laterally to the lower gluteal region and was in contact with the left lateral wall of the anal canal. No vascularity was observed. The underlying gluteal muscles appeared normal, with no evidence of hypoechoic or fistulous tracts. The right gluteal region and ischiorectal fossa were normal. Based on these findings, a diagnosis of a large perianal abscess in the left ischiorectal fossa and gluteal region was made.

## RESULTS

Surgical excision was planned, and the patient was placed in the lithotomy position for the procedure. A radial incision was made over the swelling such that the lower end of the incision was away from the external anal sphincter. The anal canal and rectum were free from the mass, and a large cyst measuring 11x8x5.5 cm was excised in toto. The defect in the levator ani was closed, and a drain was placed in situ. The postoperative period was uneventful, and the patient recovered successfully.

The specimen was sent for histopathological examination and was reported as a single grey-brown tissue mass measuring 11x8x5.5 cm (Figure 1), with the cut section showing abundant grey-brown friable material.

**Figure 1.**
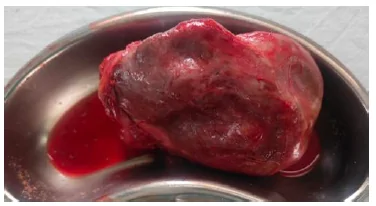
Gross specimen of the excised giant dermoid cyst from the left ischiorectal fossa, showing a well-encapsulated, lobulated mass with a smooth external surface.

Microscopy revealed that the cyst wall was lined by squamous epithelium with underlying smooth muscle fibers and fibroconnective tissue showing congested vessels (Figure 2a), as well as abundant lamellated keratin (Figure 2b), confirming the diagnosis of a dermoid cyst. Verhoeff-Van Gieson staining was used to demonstrate the smooth muscle fibers (Figure 3). The patient signed the Informed Consent for this publication.

**Figure 2.**
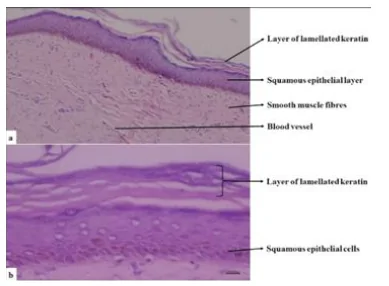
Hematoxylin and eosin-stained sections showing the cyst wall formed by squamous epithelium and the cyst lumen containing keratin (magnification: 40x).

**Figure 3.**
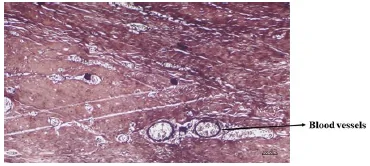
Verhoeff-Van Gieson-stained section showing blood vessels (magnification: 40x).

## DISCUSSION

The ischiorectal fossa is bounded medially by the levator ani, laterally by the fascial layer of the obturator internus, posteriorly by the lower border of the gluteus maximus and sacrotuberous ligament, and inferiorly by the skin of the perineum. It is considered the largest of the pararectal spaces^1^ ^,^ ^12^.

Although teratomas have been found in midline or paraxial structures such as the mediastinum, retroperitoneum, and other intracranial and intraspinal sites, most of them are found in the sacrococcygeal region of infants, the ovaries of adolescent females, and the testes of young males^6^. While definitive studies on its incidence are lacking, a reported prevalence of approximately 15.4-58.5% of skull and scalp masses in pediatric population has been noted, with no such figures reported for cysts seen in the adult population, as the majority of them are diagnosed in early childhood before the age of five years^10^.

 There are various theories pertaining to the exact mechanism of development of these dermoid cysts, but what is known is that uncommitted germ cells are routinely sequestered in the yolk sac’s hindgut area, close to the allantois, by the end of the third week of pregnancy. In the fourth and fifth weeks of pregnancy, these germ cells travel into the embryo by ameboid migration along the dorsal mesentery of the yolk stalk, reaching the urogenital ridge. In the sixth week, the germ cells mature into spermatogonia or oogonia.

The numerous anatomic sites observed with these masses may be explained by the migratory ability of some of these uncommitted cells, which may fail to reach the gonadal ridge, become misplaced, or move abnormally. Ultimately, while maintaining their pluripotent potential, these ectopic germ cells may grow or differentiate into dermoid cysts, teratomas, or any other type of germ cell neoplasia^6^.

Only a few cases have been reported in the literature, and the documented cases are summarized in Table 1.

**Table 1.** Cases of dermoid cysts in the ischiorectal fossa recorded in the literature.

Author	Age of the patient	Sex	Clinical features	CT/MRI findings	Ultrasound findings	Histopathology findings	Treatment
Choudur et al.^1^	55	Male	Painful right buttock and right buttock fullness for many months	A well-circumscribed right ischiorectal fossa mass (8×6×11 cm) displaced the rectum leftward. It had a thin, smooth wall without edema or osseous invasion. T1 images showed heterogeneous fat and debris; STIR revealed homogeneous fat suppression. A dense, rounded fat core with low T1/T2 signals was noted, without gadolinium enhancement.	A large, heterogeneous mass that was mostly echogenic. Streaks of posterior acoustic shadowing were observed to originate from the central ball of hyperechogenicity.	Cavity lined by squamous epithelium and filled with keratin and hair, with fibro-adipose tissue.	Complete surgical resection was performed, with no recurrence or complication reported 24 months after surgery.
Lukanovic and Patrelli^6^	24	Female	Mild pelvic pain, pelvic pressure, hesitancy of micturition, and constipation with a bulging lower gluteal region on the left side.	An oval, well-defined mass with fluid, soft tissue, and lipid densities occupied the left ischiorectal fossa, displacing the uterus rightward and posteriorly. It compressed the rectum and anus, contacting the internal obturator laterally and gluteal muscle posteriorly. Measuring 10 cm cranio-caudally, it extended from the pelvis to subcutaneous gluteal fat.	-	Cyst filled with hair and sebaceous material and lined by a thin dermis-like wall containing sebaceous glands and other skin adnexa; no immature elements were present.	The tumor was completely excised.
Kapadiya et al.^4^	32	Female	Gradual swelling and discomfort, with no signs of infection	Well-defined cystic lesion in the left ischiorectal fossa with no solid components or enhancement	-----	Stratified squamous epithelium, with no hair/teeth/bone elements	Complete surgical excision
Present case	25	Female	Presented with discomfort and a large swelling in the left gluteal region for one year, initially small and gradually progressive, which was not associated with any pain or discharge.	-	A well-defined, thick-walled collection measuring 10.4 × 9 × 9.4 cm was seen in the left ischiorectal fossa, beside the anal canal, extending to the lower gluteal region. It showed uniform low-level echoes, with no vascularity. The gluteal muscles were normal, and no fistulous tracts were detected.	The cyst wall was lined by squamous epithelium with underlying smooth muscle fibers and abundant lamellated keratin.	Surgical excision was planned, and the cyst was excised in toto. The postoperative period was uneventful, and the patient recovered well.

In the present case report, the patient presented with discomfort and a large swelling in the left gluteal region for one year. The swelling was initially small, gradually enlarged, and was not associated with pain or discharge. There was no fever related to the swelling. In a previously reported case of a retroperitoneal mass extending into the ischiorectal fossa in a nulliparous woman, symptoms included mild pelvic pain, pelvic pressure, hesitancy of micturition, and constipation^4^. In another case, a 55-year-old man presented with severe right buttock pain and fullness. On clinical examination, the overlying skin was inflamed, and severe pain prevented the patient from sitting^1^.

As dermoid cysts can be found anywhere in the body, the vast majority are found in the head and neck region, with the major presenting complaint being a painless, slow-growing mass or swelling, which can cause compressive symptoms that prompt patients to seek medical evaluation^8^.

The various differential diagnoses of ischiorectal masses include developmental malformations, such as Gartner duct cysts and tailgut cysts; inflammatory/infectious lesions, such as genitourinary infections; hematomas; and neoplastic processes, including angiomyxoma, lymphoma, liposarcoma, and lipoma with fat necrosis^3^. In this particular case, it is difficult to ascertain the exact mechanism of development or even the most likely contributing factors for the development of this lesion.

When it comes to space-occupying lesions in the ischiorectal fossa, the mainstay of the workup is radiological, with ultrasound, X-ray, computed tomography (CT), or magnetic resonance imaging (MRI) used to arrive at the various differential diagnoses. In the present case, ultrasonography revealed homogeneous, low-level thick echoes, which aided in the diagnosis of a benign cystic lesion^5^. Distinctive histopathological features are crucial for making a definitive diagnosis, thereby emphasizing that radiological examination alone cannot be used to establish the diagnosis. When observed under a microscope, the key features of dermoid cysts are well-circumscribed, encapsulated lesions lined by squamous epithelium derived from the ectoderm and containing various cutaneous appendages (such as sebaceous glands, sweat glands, or hair follicles) and a cyst lumen containing keratin, as seen in the present case, and sometimes sebaceous material and occasionally hair^1^.

Additionally, abnormally high AFP, CEA, CA 19-9, and CA 125 levels were observed in retroperitoneal teratomas. Elevated serum markers are more common in patients with malignancy than in those with immature or benign lesions^7^.

Surgical excision was performed in the present case to prevent the benign lesion from progressing into a fistula or undergoing malignant transformation. Dermoid cysts occurring in the ischiorectal fossa are extremely rare, and in this case, there was no evidence of hair or bone within the cyst. In these cases, along with ultrasound, MRI findings depict the extent of the lesion, which helps guide surgical treatment^3^ ^,^ ^9^. Owing to the possibility of rupture, infection, and malignancy, a large dermoid cyst, such as the one in the present case, needs to be surgically excised.

## CONCLUSIONS

This case highlights the rare presentation of a giant dermoid cyst in the left ischiorectal fossa of a young female patient, emphasizing the importance of considering such uncommon lesions in the differential diagnosis of perineal and pelvic masses. Early imaging and surgical excision are crucial for accurate diagnosis, effective management, and prevention of potential complications of this condition. To prevent injury to adjacent structures, such as the bladder, urethra, rectum, and anal canal, careful preoperative assessment and meticulous dissection are essential, supported by a thorough understanding of the complex anatomy of the ischiorectal fossa. This report contributes to the limited literature on dermoid cysts in this unusual anatomical location.

## Data Availability

The datasets generated and/or analyzed during the current study are available from the corresponding author upon reasonable request.
